# Supplementary material for: End Sequence Analysis Toolkit (ESAT) expands the extractable information from single-cell RNA-seq data
Source: Genome Res. 2016 Oct;26(10):1397–410. doi: 10.1101/gr.207902.116 (PMC5052061; doi:10.1101/gr.207902.116)
Supplement: Supplemental Material [file supp_gr.207902.116_Supp_Materials.tar.gz › ESAT-esat_v0.1/doc/html/classumms_1_1core_1_1annotation_1_1_gene.html]

ESAT: umms.core.annotation.Gene Class Reference


ESAT


- Main Page
- Related Pages
- Packages
- Classes
- Files

- Class List
- Class Index
- Class Hierarchy
- Class Members

All Classes Namespaces Files Functions Variables Enumerator Pages

- umms
- core
- annotation
- Gene

Public Member Functions |
Static Public Member Functions |
Static Public Attributes |
Protected Member Functions |
List of all members

umms.core.annotation.Gene Class Reference

Inheritance diagram for umms.core.annotation.Gene:

[legend]

Collaboration diagram for umms.core.annotation.Gene:

[legend]

|  |  |
| --- | --- |
| Public Member Functions | |
|  | Gene (String chr, String name, Strand orientation, Collection<?extends Annotation > exons, int cdsStart, int cdsEnd) |
|  | |
|  | Gene (String chr, int start, int end, String name, Strand orientation, int cdsStart, int cdsEnd) |
|  | |
|  | Gene (Collection<?extends Annotation > exons, String name, Strand orientation, int cdsStart, int cdsEnd) |
|  | |
|  | Gene (String chr, String name, Strand orientation, Collection<?extends Annotation > exons) |
|  | |
|  | Gene (Annotation annotation, String name, Strand orientation, Collection<?extends Annotation > exons) |
|  | |
|  | Gene (Annotation annotation, Collection<?extends Annotation > exons) |
|  | |
|  | Gene (Annotation annotation, Collection<?extends Annotation > exons, String name, int cdsStart, int cdsEnd) |
|  | |
|  | Gene (Annotation annotation, Collection<?extends Annotation > exons, String name) |
|  | |
|  | Gene (Collection<?extends Annotation > exons, String name, Strand orientation) |
|  | |
|  | Gene (String chr, int start, int end, String name, Strand orientation) |
|  | |
|  | Gene (String chr, int start, int end, String name) |
|  | |
|  | Gene (Collection<?extends Annotation > exons, String name) |
|  | |
|  | Gene (Collection<?extends Annotation > exons) |
|  | |
|  | Gene (String chr, int start, int end) |
|  | |
|  | Gene (Alignments align) |
|  | |
|  | Gene (Annotation annotation) |
|  | |
|  | Gene (String pslString) |
|  | |
|  | Gene (String pslString, boolean isPSLFormat) |
|  | |
|  | Gene (String chr2, int start2, int end, String name2, double bedScore2, String orientation2, String sequence2, String[] extraFields2) |
|  | |
|  | Gene (String chr2, int start2, int end, String name2, double bedScore2, String orientation2, String sequence2) |
|  | |
|  | Gene (String chr, int start, int end, String name, double mybedScore, String orientation, int[] exonsStart, int[] exonsEnd) |
|  | |
|  | Gene (String chr, int start, int end, String name, String orientation, List< Integer > exonsStart, List< Integer > exonsEnd) |
|  | |
|  | Gene (String chr, int start, int end, String name, String orientation, List< Integer > exonsStart, List< Integer > exonsEnd, int blockStart, int blockEnd) |
|  | |
|  | Gene (String chr, int start, int end, String name, String orientation, List< Integer > exonsStart, List< Integer > exonsEnd, String[] extraData) |
|  | |
|  | Gene (String chr2, int start2, int end, String name2, double bedScore2, String orientation2, int[] exonStarts2, int[] exonEnds2, double[] exonScores2, String sequence2) |
|  | |
|  | Gene (String chr2, int start2, int end, String name2, double bedScore2, String orientation2, int[] exonStarts2, int[] exonEnds2, double[] exonScores2, String sequence2, String[] extraFields2) |
|  | |
|  | Gene (String chr, int start, int end, String name, double score, String strand, List< Integer > exonsStart, List< Integer > exonsEnd, int blockStart2, int blockEnd2) |
|  | |
|  | Gene (String chr, int start, int end, String name, String orientation, List< Integer > exonsStart, List< Integer > exonsEnd, int blockStart, int blockEnd, String[] extraData) |
|  | |
|  | Gene (String chr, int start, int end, String name, double score, String strand, List< Integer > exonsStart, List< Integer > exonsEnd, int blockStart2, int blockEnd2, String[] extraColumns) |
|  | |
|  | Gene (Gene gene) |
|  | |
| void | setSequence (String seq) |
|  | |
| void | setSequenceFromChromosome (Sequence chrSequence) |
|  | |
| void | setOrientation (String orientation) |
|  | |
| void | setSAMString (String sam) |
|  | |
| void | setExtraFields (double[] d) |
|  | |
| void | setExtraFields (double val, int i) |
|  | |
| void | setExtraFields (String[] extraData) |
|  | |
| int | getCDSEnd () |
|  | |
| int | getCDSStart () |
|  | |
| void | setCDSRegion (int start, int end) |
|  | |
| void | setBedScore (double d) |
|  | |
| void | setCountScore (double score) |
|  | |
| int | getSize () |
|  | |
| int | getGenomicLength () |
|  | |
| boolean | isNegativeStrand () |
|  | |
| String | getSequence () |
|  | |
| Sequence | getSequence (Sequence chrSequence) |
|  | |
| String | getSequence (Sequence chr2, boolean repeatMask, boolean stranded) throws Exception |
|  | |
| Sequence | getSequenceObject () |
|  | |
| String | getString () |
|  | |
| String | getSAMString () |
|  | |
| String | getAttribute (String name) |
|  | |
| Map | getAttributes () |
|  | |
| String[] | getExtraFields () |
|  | |
| String | getExtraFields (int i) |
|  | |
| double | getNormalizedScore () |
|  | |
| double | getRPKM () |
|  | |
| double | getPValue () |
|  | |
| double | getCountScore () |
|  | |
| double[] | getExonsScores () |
|  | |
| int | getTranscriptLength () |
|  | |
| Collection<?extends Annotation > | get5Utr () |
|  | |
| Collection<?extends Annotation > | get3Utr () |
|  | |
| Gene | get3UTRGene () |
|  | |
| Gene | get5UTRGene () |
|  | |
| Annotation | get5UTR () |
|  | |
| Annotation | get3UTR () |
|  | |
| boolean | hasCDS () |
|  | |
| Iterator< Integer > | getAllPositions () |
|  | |
| Collection< GeneWindow > | getWindows (int windowSize) |
|  | |
| Collection< GeneWindow > | getWindows (int windowSize, int stepSize, int start) |
|  | |
| Gene | getStartCodon () |
|  | |
| Annotation | get5UtrIncludingIntrons () |
|  | |
| String | getChrNum () |
|  | |
| Alignments | get3UtrIncludingIntrons () |
|  | |
| int | getNumExons () |
|  | |
| Alignments[] | getExons () |
|  | |
| Set<?extends Annotation > | getExonSet () |
|  | |
| Collection<?extends Annotation > | getSortedAndUniqueExons () |
|  | |
| IntervalTree< Annotation > | getExonTree () |
|  | |
| Annotation | getFirstExon () |
|  | |
| Annotation | getLastExon () |
|  | |
| Annotation | getOrientedLastExon () |
|  | |
| Annotation | get5PrimeExon () |
|  | |
| Annotation | get3PrimeExon (int size) |
|  | |
| Annotation | get3PrimeExon () |
|  | |
| Gene | getIntrons () |
|  | |
| Annotation | getLastIntron () |
|  | |
| Annotation | getFirstIntron () |
|  | |
| Alignments[] | getIntronsBlocks () |
|  | |
| Collection<?extends Annotation > | getIntronSet () |
|  | |
| Gene | getIntronTranscript () |
|  | |
| boolean | isCodingGene () |
|  | |
| Gene | getCDS () |
|  | |
| Alignments | getCDSRegion () |
|  | |
| Annotation | getAlignment () |
|  | |
| Alignments | getPromoter (int fudgeFactor) |
|  | |
| Alignments | getPromoter (int upstream, int downstream) |
|  | |
| double | getNumberPositions (int rate) |
|  | |
| int | getGappedSize () |
|  | |
| double | getBedScore () |
|  | |
| int | get3PrimeGenomicDistance (LightweightGenomicAnnotation annot) |
|  | |
| Gene | getOverlap (Gene second) |
|  | |
| Gene | getOverlap (Collection< Gene > others) |
|  | |
| ArrayList< Integer > | getSpliceJunctionCoordinates () throws Exception |
|  | |
| IntervalTree< Integer > | getSpliceJunctionCoordinatesTree () throws Exception |
|  | |
| String | toString () |
|  | |
| String | toRefSeq () |
|  | |
| String | toUCSC () |
|  | |
| String | toBED () |
|  | |
| String | toBED (int r, int g, int b) |
|  | |
| String | toBED (boolean useExtraFields) |
|  | |
| String | toBED (boolean useExtraFields, int r, int g, int b) |
|  | |
| String | toBEDwithBedScore () |
|  | |
| String | toGTF (String source) |
|  | |
| String | toGTF (String source, String geneName, String transcriptName) |
|  | |
| String | toCufflinksGTF (String source, String geneName, String transcriptName, String nonParsedAttrs) |
|  | |
| String | toSAM () |
|  | |
| String | toSAM (String originalSAMLine) |
|  | |
| String | toSAM (String originalSAMLine, boolean flip) |
|  | |
| void | setCDS (Annotation cdsRegion) |
|  | |
| int | absoluteToRelativePosition (int referenceCoordinate) |
|  | |
| boolean | isInExon (int snp) |
|  | |
| Collection< Gene > | findAllORFs (boolean trim3UTRs) |
|  | |
| TreeSet  < BasicLightweightAnnotation > | findAllStartCodons () |
|  | |
| Collection< Gene > | findAllORFs (Sequence chrSeq, boolean trim3UTRs) |
|  | |
| void | addSequence (Sequence seq) |
|  | |
| void | addSuffixToName (String refName) |
|  | |
| void | addAttribute (String name, String val) |
|  | |
| void | addExtraField (String value) |
|  | |
| void | expandUtrs (Integer utr1, Integer utr2) |
|  | |
| boolean | geneSpanContains (Gene gene) |
|  | |
| boolean | containsAttribute (String key) |
|  | |
| boolean | isFullyCompatible (Gene iso) |
|  | |
| boolean | overlapsExon (Annotation gen) |
|  | |
| boolean | overlapsExon (LightweightGenomicAnnotation exon) |
|  | |
| boolean | overlaps (Gene other) |
|  | |
| int | distanceToNearestNonOverlapper (Collection< Annotation > others) |
|  | |
| boolean | overlaps (Gene other, boolean ignoreOrientation) |
|  | |
| boolean | overlaps (Gene other, double minPctOverlap) |
|  | |
| boolean | overlapsStranded (Gene other, double minPctOverlap) |
|  | |
| boolean | overlapsGene (Collection< Gene > others) |
|  | |
| boolean | overlapsGene (Gene gen) |
|  | |
| boolean | overlapsGeneInAnyOrientation (Gene otherGene) |
|  | |
| boolean | overlaps2 (LightweightGenomicAnnotation next) |
|  | |
| boolean | almostEqual (Gene other, int exonFudgeFactor) |
|  | |
| boolean | almostSameStructure (Gene other, int intronFudgeFactor) |
|  | |
| boolean | almostContains (Gene other, int exonFudgeFactor) |
|  | |
| int | hashCode () |
|  | |
| int | compareTo (Annotation other) |
|  | |
| Gene | copy () |
|  | |
| GeneWindow | trimGene (int relativeStart, int relativeEnd) |
|  | |
| void | setCDSStart (int start) |
|  | |
| void | setCDSEnd (int end) |
|  | |
| Gene | findLongestORF () |
|  | |
| int | numOverlappingExons (Gene iso) |
|  | |
| int | numOfCompatibleIntrons (Gene g) |
|  | |
| GeneWindow | trimAbsolute (int alignmentStart, int alignmentEnd) |
|  | |
| double | percentOverlapping (Gene gene2) |
|  | |
| Double | percentGenomeOverlapping (Gene gene2) |
|  | |
| int | transcriptToGenomicPosition (int transcriptPosition) |
|  | |
| int | getMidpointGenomicCoords () |
|  | |
| Gene | transcriptToGenomicPosition (int startPosOnTranscript, int endPosOnTranscript) |
|  | |
| int | genomicToTranscriptPosition (int genomicPosition) |
|  | |
| int | genomicToGenomicPositionWithOffset (int genomicPosition, int offset) |
|  | |
| Gene | takeUnion (Gene other) |
|  | |
| double | getPercentCDS () |
|  | |
| double | getPercent3UTR () |
|  | |
| Gene | extendAnnotation (int deltaStart, int deltaEnd) |
|  | |
| void | addIsoform (Gene g) |
|  | |
| Collection< Gene > | getIsoforms () |
|  | |
| Public Member Functions inherited from umms.core.annotation.BasicAnnotation | |
|  | BasicAnnotation () |
|  | |
|  | BasicAnnotation (SAMRecord samRecord) |
|  | |
|  | BasicAnnotation (String ucsc) |
|  | |
|  | BasicAnnotation (String referenceName, int start, int end, Strand orientation, String name) |
|  | |
|  | BasicAnnotation (String referenceName, Strand orientation, String name, Collection<?extends Annotation > blocks) |
|  | |
|  | BasicAnnotation (String referenceName, int start, int end) |
|  | |
|  | BasicAnnotation (String referenceName, int start, int end, Strand orientation) |
|  | |
|  | BasicAnnotation (String referenceName, int start, int end, String orientation) |
|  | |
|  | BasicAnnotation (BasicAnnotation other) |
|  | |
|  | BasicAnnotation (Annotation other) |
|  | |
|  | BasicAnnotation (Collection<?extends Annotation > blocks) |
|  | |
|  | BasicAnnotation (Collection<?extends Annotation > blocks, Strand orientation, String name) |
|  | |
|  | BasicAnnotation (Collection<?extends Annotation > blocks, String name) |
|  | |
|  | BasicAnnotation (String referenceName, CompoundInterval blocks, Strand orientation, String name) |
|  | |
|  | BasicAnnotation (String referenceName, CompoundInterval blocks, Strand orientation) |
|  | |
|  | BasicAnnotation (String referenceName, CompoundInterval blocks) |
|  | |
| Annotation | copy () |
|  | |
| int | getStart () |
|  | |
| int | getEnd () |
|  | |
| int | getMidpoint () |
|  | |
| String | getReferenceName () |
|  | |
| String | getName () |
|  | |
| Strand | getOrientation () |
|  | |
| boolean | isUnoriented () |
|  | |
| List<?extends Annotation > | getBlocks () |
|  | |
| List<?extends Annotation > | getBlocks (boolean oriented) |
|  | |
| Annotation[] | getFlankingBlocks (Annotation spliceJunction) |
|  | |
| int | numBlocks () |
|  | |
| int | length () |
|  | |
| int | getReferenceCoordinateAtPosition (int positionInAnnotation, boolean ignoreOrientation) |
|  | |
| int | getPositionAtReferenceCoordinate (int referenceCoordinate, boolean ignoreOrientation) |
|  | |
| double | getScore () |
|  | |
| String | toString () |
|  | |
| void | setStart (int start) |
|  | |
| void | setEnd (int end) |
|  | |
| void | setOrientation (Strand orientation) |
|  | |
| void | setOrientedStart (int orientedStart) |
|  | |
| void | setOrientedEnd (int orientedEnd) |
|  | |
| void | setReferenceName (String refName) |
|  | |
| void | setName (String n) |
|  | |
| void | setScore (double s) |
|  | |
| void | addBlocks (Annotation block) |
|  | |
| void | addBlocks (Collection<?extends Annotation > blocks) |
|  | |
| void | shift (int delta) |
|  | |
| void | moveToCoordinate (int coordinateInReference) |
|  | |
| boolean | overlaps (Annotation other, int buffer, boolean considerOrientation) |
|  | |
| boolean | overlaps (List<?extends Annotation > otherBlocks, int buffer) |
|  | |
| int | getOverlap (Annotation other) |
|  | |
| boolean | contains (Annotation other) |
|  | |
| Annotation | union (Annotation other) |
|  | |
| Annotation | intersect (Annotation other) |
|  | |
| List< Annotation > | disect (Annotation a) |
|  | |
| List< Annotation > | disect (List<?extends Annotation > disectors) |
|  | |
| Annotation | minus (Annotation other) |
|  | |
| Annotation | minus (Collection<?extends Annotation > others) |
|  | |
| void | stitchTo (Annotation next) |
|  | |
| Annotation | complement () |
|  | |
| Collection<?extends Annotation > | getSpliceConnections () |
|  | |
| Public Member Functions inherited from umms.core.annotation.AbstractAnnotation | |
| int | size () |
|  | |
| int | getSize () |
|  | |
| int | getMidpoint () |
|  | |
| Strand | getStrand () |
|  | |
| boolean | hasOrientation () |
|  | |
| boolean | isNegativeStrand () |
|  | |
| String | getChr () |
|  | |
| int | getOrientedStart () |
|  | |
| int | getOrientedEnd () |
|  | |
| int | getLengthOnReference () |
|  | |
| int | getReferenceCoordinateAtPosition (int positionInAnnotation) |
|  | |
| int | getPositionAtReferenceCoordinate (int referenceCoordinate) |
|  | |
| void | setOrientation (char orientation) |
|  | |
| void | expand (int deltaStart, int deltaEnd) |
|  | |
| Annotation | trim (int deltaStart, int deltaEnd) |
|  | |
| boolean | fullyContains (Annotation other) |
|  | |
| boolean | overlaps (Annotation other) |
|  | |
| boolean | overlaps (Annotation other, int buffer) |
|  | |
| boolean | overlapsStranded (Annotation other) |
|  | |
| boolean | overlaps (Annotation other, boolean considerOrientation) |
|  | |
| boolean | overlaps (Collection<?extends Annotation > others) |
|  | |
| boolean | overlaps (Collection<?extends Annotation > others, int buffer) |
|  | |
| String | toUCSC () |
|  | |
| String | toBED () |
|  | |
| final String | getFullInfoString () |
|  | |
| String | toBED (int r, int g, int b) |
|  | |
| String | toShortBED () |
|  | |
| String | toBEDGraph () |
|  | |
| int | getDistanceTo (Annotation other) |
|  | |
| String | toCigar () |
|  | |
| List< Annotation > | intersect (List<?extends Annotation > others) |
|  | |
| int | compareTo (Annotation other) |
|  | |
| int | compareToAnnotation (Annotation b) |
|  | |
| int | compareToAnnotation (Annotation b, boolean useOrientation) |
|  | |
| boolean | equals (Annotation a) |
|  | |
| boolean | equals (Annotation a, boolean useOrientation) |
|  | |
| int | getSAMStart () |
|  | |
| int | getSAMEnd () |
|  | |

|  |  |
| --- | --- |
| Static Public Member Functions | |
| static Collection< int[]> | findAllORFs (String sequence) |
|  | |
| static boolean | isStopCodon (String currentCodon) |
|  | |
| static Collection< Gene > | getOverlappers (Collection< Gene > genes, String chr, int start, int end, Strand strand, boolean ignoreOrientation) |
|  | |
| static int[] | findLongestORF (String sequence) |
|  | |
| Static Public Member Functions inherited from umms.core.annotation.BasicAnnotation | |
| static Annotation | createFromUCSC (String ucsc) |
|  | |
| static BasicAnnotation | fromFullInfoString (String fullInfoString) |
|  | |
| static void | main (String[] args) throws IOException |
|  | |
| Static Public Member Functions inherited from umms.core.annotation.AbstractAnnotation | |
| static Strand | getStrand (String orientation) |
|  | |
| static Strand | getStrand (char orientation) |
|  | |

|  |  |
| --- | --- |
| Static Public Attributes | |
| static final Pattern | START\_CODON\_PATRN = Pattern.compile("ATG", Pattern.CASE\_INSENSITIVE) |
|  | |
| static final String[] | STOP\_CODONS = {"TAG", "TAA", "TGA"} |
|  | |
| Static Public Attributes inherited from umms.core.annotation.AbstractAnnotation | |
| static final int | MAX\_DISTANCE = 1000000000 |
|  | |

|  |  |
| --- | --- |
| Protected Member Functions | |
| void | initFromGene (Gene gene) |
|  | |
| boolean | almostContainsRefSeq (Gene other, int exonFudgeFactor) |
|  | |

|  |  |
| --- | --- |
| Additional Inherited Members | |
| Protected Attributes inherited from umms.core.annotation.BasicAnnotation | |
| CompoundInterval | blocks = new CompoundInterval() |
|  | |

## Constructor & Destructor Documentation

|  |  |  |  |
| --- | --- | --- | --- |
| umms.core.annotation.Gene.Gene | ( | String | *chr*, |
|  |  | String | *name*, |
|  |  | Strand | *orientation*, |
|  |  | Collection<?extends Annotation > | *exons*, |
|  |  | int | *cdsStart*, |
|  |  | int | *cdsEnd* |
|  | ) |  |  |

Here is the caller graph for this function:

|  |  |  |  |
| --- | --- | --- | --- |
| umms.core.annotation.Gene.Gene | ( | String | *chr*, |
|  |  | int | *start*, |
|  |  | int | *end*, |
|  |  | String | *name*, |
|  |  | Strand | *orientation*, |
|  |  | int | *cdsStart*, |
|  |  | int | *cdsEnd* |
|  | ) |  |  |

|  |  |  |  |
| --- | --- | --- | --- |
| umms.core.annotation.Gene.Gene | ( | Collection<?extends Annotation > | *exons*, |
|  |  | String | *name*, |
|  |  | Strand | *orientation*, |
|  |  | int | *cdsStart*, |
|  |  | int | *cdsEnd* |
|  | ) |  |  |

|  |  |  |  |
| --- | --- | --- | --- |
| umms.core.annotation.Gene.Gene | ( | String | *chr*, |
|  |  | String | *name*, |
|  |  | Strand | *orientation*, |
|  |  | Collection<?extends Annotation > | *exons* |
|  | ) |  |  |

|  |  |  |  |
| --- | --- | --- | --- |
| umms.core.annotation.Gene.Gene | ( | Annotation | *annotation*, |
|  |  | String | *name*, |
|  |  | Strand | *orientation*, |
|  |  | Collection<?extends Annotation > | *exons* |
|  | ) |  |  |

|  |  |  |  |
| --- | --- | --- | --- |
| umms.core.annotation.Gene.Gene | ( | Annotation | *annotation*, |
|  |  | Collection<?extends Annotation > | *exons* |
|  | ) |  |  |

Here is the call graph for this function:

|  |  |  |  |
| --- | --- | --- | --- |
| umms.core.annotation.Gene.Gene | ( | Annotation | *annotation*, |
|  |  | Collection<?extends Annotation > | *exons*, |
|  |  | String | *name*, |
|  |  | int | *cdsStart*, |
|  |  | int | *cdsEnd* |
|  | ) |  |  |

Here is the call graph for this function:

|  |  |  |  |
| --- | --- | --- | --- |
| umms.core.annotation.Gene.Gene | ( | Annotation | *annotation*, |
|  |  | Collection<?extends Annotation > | *exons*, |
|  |  | String | *name* |
|  | ) |  |  |

Here is the call graph for this function:

|  |  |  |  |
| --- | --- | --- | --- |
| umms.core.annotation.Gene.Gene | ( | Collection<?extends Annotation > | *exons*, |
|  |  | String | *name*, |
|  |  | Strand | *orientation* |
|  | ) |  |  |

|  |  |  |  |
| --- | --- | --- | --- |
| umms.core.annotation.Gene.Gene | ( | String | *chr*, |
|  |  | int | *start*, |
|  |  | int | *end*, |
|  |  | String | *name*, |
|  |  | Strand | *orientation* |
|  | ) |  |  |

|  |  |  |  |
| --- | --- | --- | --- |
| umms.core.annotation.Gene.Gene | ( | String | *chr*, |
|  |  | int | *start*, |
|  |  | int | *end*, |
|  |  | String | *name* |
|  | ) |  |  |

|  |  |  |  |
| --- | --- | --- | --- |
| umms.core.annotation.Gene.Gene | ( | Collection<?extends Annotation > | *exons*, |
|  |  | String | *name* |
|  | ) |  |  |

|  |  |  |  |  |  |
| --- | --- | --- | --- | --- | --- |
| umms.core.annotation.Gene.Gene | ( | Collection<?extends Annotation > | *exons* | ) |  |

|  |  |  |  |
| --- | --- | --- | --- |
| umms.core.annotation.Gene.Gene | ( | String | *chr*, |
|  |  | int | *start*, |
|  |  | int | *end* |
|  | ) |  |  |

|  |  |  |  |  |  |
| --- | --- | --- | --- | --- | --- |
| umms.core.annotation.Gene.Gene | ( | Alignments | *align* | ) |  |

Here is the call graph for this function:

|  |  |  |  |  |  |
| --- | --- | --- | --- | --- | --- |
| umms.core.annotation.Gene.Gene | ( | Annotation | *annotation* | ) |  |

Here is the call graph for this function:

|  |  |  |  |  |  |
| --- | --- | --- | --- | --- | --- |
| umms.core.annotation.Gene.Gene | ( | String | *pslString* | ) |  |

|  |  |  |  |
| --- | --- | --- | --- |
| umms.core.annotation.Gene.Gene | ( | String | *pslString*, |
|  |  | boolean | *isPSLFormat* |
|  | ) |  |  |

|  |  |  |  |
| --- | --- | --- | --- |
| umms.core.annotation.Gene.Gene | ( | String | *chr2*, |
|  |  | int | *start2*, |
|  |  | int | *end*, |
|  |  | String | *name2*, |
|  |  | double | *bedScore2*, |
|  |  | String | *orientation2*, |
|  |  | String | *sequence2*, |
|  |  | String[] | *extraFields2* |
|  | ) |  |  |

|  |  |  |  |
| --- | --- | --- | --- |
| umms.core.annotation.Gene.Gene | ( | String | *chr2*, |
|  |  | int | *start2*, |
|  |  | int | *end*, |
|  |  | String | *name2*, |
|  |  | double | *bedScore2*, |
|  |  | String | *orientation2*, |
|  |  | String | *sequence2* |
|  | ) |  |  |

|  |  |  |  |
| --- | --- | --- | --- |
| umms.core.annotation.Gene.Gene | ( | String | *chr*, |
|  |  | int | *start*, |
|  |  | int | *end*, |
|  |  | String | *name*, |
|  |  | double | *mybedScore*, |
|  |  | String | *orientation*, |
|  |  | int[] | *exonsStart*, |
|  |  | int[] | *exonsEnd* |
|  | ) |  |  |

|  |  |  |  |
| --- | --- | --- | --- |
| umms.core.annotation.Gene.Gene | ( | String | *chr*, |
|  |  | int | *start*, |
|  |  | int | *end*, |
|  |  | String | *name*, |
|  |  | String | *orientation*, |
|  |  | List< Integer > | *exonsStart*, |
|  |  | List< Integer > | *exonsEnd* |
|  | ) |  |  |

|  |  |  |  |
| --- | --- | --- | --- |
| umms.core.annotation.Gene.Gene | ( | String | *chr*, |
|  |  | int | *start*, |
|  |  | int | *end*, |
|  |  | String | *name*, |
|  |  | String | *orientation*, |
|  |  | List< Integer > | *exonsStart*, |
|  |  | List< Integer > | *exonsEnd*, |
|  |  | int | *blockStart*, |
|  |  | int | *blockEnd* |
|  | ) |  |  |

|  |  |  |  |
| --- | --- | --- | --- |
| umms.core.annotation.Gene.Gene | ( | String | *chr*, |
|  |  | int | *start*, |
|  |  | int | *end*, |
|  |  | String | *name*, |
|  |  | String | *orientation*, |
|  |  | List< Integer > | *exonsStart*, |
|  |  | List< Integer > | *exonsEnd*, |
|  |  | String[] | *extraData* |
|  | ) |  |  |

Here is the call graph for this function:

|  |  |  |  |
| --- | --- | --- | --- |
| umms.core.annotation.Gene.Gene | ( | String | *chr2*, |
|  |  | int | *start2*, |
|  |  | int | *end*, |
|  |  | String | *name2*, |
|  |  | double | *bedScore2*, |
|  |  | String | *orientation2*, |
|  |  | int[] | *exonStarts2*, |
|  |  | int[] | *exonEnds2*, |
|  |  | double[] | *exonScores2*, |
|  |  | String | *sequence2* |
|  | ) |  |  |

|  |  |  |  |
| --- | --- | --- | --- |
| umms.core.annotation.Gene.Gene | ( | String | *chr2*, |
|  |  | int | *start2*, |
|  |  | int | *end*, |
|  |  | String | *name2*, |
|  |  | double | *bedScore2*, |
|  |  | String | *orientation2*, |
|  |  | int[] | *exonStarts2*, |
|  |  | int[] | *exonEnds2*, |
|  |  | double[] | *exonScores2*, |
|  |  | String | *sequence2*, |
|  |  | String[] | *extraFields2* |
|  | ) |  |  |

|  |  |  |  |
| --- | --- | --- | --- |
| umms.core.annotation.Gene.Gene | ( | String | *chr*, |
|  |  | int | *start*, |
|  |  | int | *end*, |
|  |  | String | *name*, |
|  |  | double | *score*, |
|  |  | String | *strand*, |
|  |  | List< Integer > | *exonsStart*, |
|  |  | List< Integer > | *exonsEnd*, |
|  |  | int | *blockStart2*, |
|  |  | int | *blockEnd2* |
|  | ) |  |  |

|  |  |  |  |
| --- | --- | --- | --- |
| umms.core.annotation.Gene.Gene | ( | String | *chr*, |
|  |  | int | *start*, |
|  |  | int | *end*, |
|  |  | String | *name*, |
|  |  | String | *orientation*, |
|  |  | List< Integer > | *exonsStart*, |
|  |  | List< Integer > | *exonsEnd*, |
|  |  | int | *blockStart*, |
|  |  | int | *blockEnd*, |
|  |  | String[] | *extraData* |
|  | ) |  |  |

|  |  |  |  |
| --- | --- | --- | --- |
| umms.core.annotation.Gene.Gene | ( | String | *chr*, |
|  |  | int | *start*, |
|  |  | int | *end*, |
|  |  | String | *name*, |
|  |  | double | *score*, |
|  |  | String | *strand*, |
|  |  | List< Integer > | *exonsStart*, |
|  |  | List< Integer > | *exonsEnd*, |
|  |  | int | *blockStart2*, |
|  |  | int | *blockEnd2*, |
|  |  | String[] | *extraColumns* |
|  | ) |  |  |

|  |  |  |  |  |  |
| --- | --- | --- | --- | --- | --- |
| umms.core.annotation.Gene.Gene | ( | Gene | *gene* | ) |  |

Here is the call graph for this function:

## Member Function Documentation

|  |  |  |  |  |  |
| --- | --- | --- | --- | --- | --- |
| int umms.core.annotation.Gene.absoluteToRelativePosition | ( | int | *referenceCoordinate* | ) |  |

Here is the call graph for this function:

|  |  |  |  |
| --- | --- | --- | --- |
| void umms.core.annotation.Gene.addAttribute | ( | String | *name*, |
|  |  | String | *val* |
|  | ) |  |  |

|  |  |  |  |  |  |
| --- | --- | --- | --- | --- | --- |
| void umms.core.annotation.Gene.addExtraField | ( | String | *value* | ) |  |

|  |  |  |  |  |  |
| --- | --- | --- | --- | --- | --- |
| void umms.core.annotation.Gene.addIsoform | ( | Gene | *g* | ) |  |

Here is the call graph for this function:

|  |  |  |  |  |  |
| --- | --- | --- | --- | --- | --- |
| void umms.core.annotation.Gene.addSequence | ( | Sequence | *seq* | ) |  |

|  |  |  |  |  |  |
| --- | --- | --- | --- | --- | --- |
| void umms.core.annotation.Gene.addSuffixToName | ( | String | *refName* | ) |  |

|  |  |  |  |
| --- | --- | --- | --- |
| boolean umms.core.annotation.Gene.almostContains | ( | Gene | *other*, |
|  |  | int | *exonFudgeFactor* |
|  | ) |  |  |

Here is the call graph for this function:

|  |  |  |  |
| --- | --- | --- | --- |
| boolean umms.core.annotation.Gene.almostContainsRefSeq | ( | Gene | *other*, |
|  |  | int | *exonFudgeFactor* |
|  | ) |  |  |

protected

Here is the call graph for this function:

Here is the caller graph for this function:

|  |  |  |  |
| --- | --- | --- | --- |
| boolean umms.core.annotation.Gene.almostEqual | ( | Gene | *other*, |
|  |  | int | *exonFudgeFactor* |
|  | ) |  |  |

Here is the call graph for this function:

|  |  |  |  |
| --- | --- | --- | --- |
| boolean umms.core.annotation.Gene.almostSameStructure | ( | Gene | *other*, |
|  |  | int | *intronFudgeFactor* |
|  | ) |  |  |

Here is the call graph for this function:

|  |  |  |  |  |  |
| --- | --- | --- | --- | --- | --- |
| int umms.core.annotation.Gene.compareTo | ( | Annotation | *other* | ) |  |

Here is the call graph for this function:

Here is the caller graph for this function:

|  |  |  |  |  |  |
| --- | --- | --- | --- | --- | --- |
| boolean umms.core.annotation.Gene.containsAttribute | ( | String | *key* | ) |  |

|  |  |  |  |  |
| --- | --- | --- | --- | --- |
| Gene umms.core.annotation.Gene.copy | ( |  | ) |  |

Returns
:   A deep clone of this Annotation object

Implements umms.core.annotation.Annotation.

Here is the call graph for this function:

Here is the caller graph for this function:

|  |  |  |  |  |  |
| --- | --- | --- | --- | --- | --- |
| int umms.core.annotation.Gene.distanceToNearestNonOverlapper | ( | Collection< Annotation > | *others* | ) |  |

Get inner distance between this gene and its nearest neighbor in a collection Ignore genes that overlap this gene in any orientation Looks at entire gene span, not just exons

Parameters
:   |  |  |
    | --- | --- |
    | others | Other genes to check |

Returns
:   Min distance to a non-overlapper in the collection, or -1 if no other genes on same chromosome

Here is the call graph for this function:

|  |  |  |  |
| --- | --- | --- | --- |
| void umms.core.annotation.Gene.expandUtrs | ( | Integer | *utr1*, |
|  |  | Integer | *utr2* |
|  | ) |  |  |

Here is the call graph for this function:

|  |  |  |  |
| --- | --- | --- | --- |
| Gene umms.core.annotation.Gene.extendAnnotation | ( | int | *deltaStart*, |
|  |  | int | *deltaEnd* |
|  | ) |  |  |

Get new gene extended in either direction

Parameters
:   |  |  |
    | --- | --- |
    | deltaStart | Distance to extend at begin position |
    | deltaEnd | Distance to extend at end position |

Returns
:   New extended gene

Here is the call graph for this function:

|  |  |  |  |  |  |
| --- | --- | --- | --- | --- | --- |
| Collection<Gene> umms.core.annotation.Gene.findAllORFs | ( | boolean | *trim3UTRs* | ) |  |

Get all ORFs as gene objects

Parameters
:   |  |  |
    | --- | --- |
    | trim3UTRs | whether to trim 3'UTRs to end at the beginning of next ORF |

Returns
:   the collection of ORFs

Here is the call graph for this function:

Here is the caller graph for this function:

|  |  |  |  |  |  |
| --- | --- | --- | --- | --- | --- |
| static Collection<int[]> umms.core.annotation.Gene.findAllORFs | ( | String | *sequence* | ) |  |

static

Here is the call graph for this function:

|  |  |  |  |
| --- | --- | --- | --- |
| Collection<Gene> umms.core.annotation.Gene.findAllORFs | ( | Sequence | *chrSeq*, |
|  |  | boolean | *trim3UTRs* |
|  | ) |  |  |

Here is the call graph for this function:

|  |  |  |  |  |
| --- | --- | --- | --- | --- |
| TreeSet<BasicLightweightAnnotation> umms.core.annotation.Gene.findAllStartCodons | ( |  | ) |  |

Find start codons

Returns
:   collection of all start codon genomic coordinates

Here is the call graph for this function:

|  |  |  |  |  |
| --- | --- | --- | --- | --- |
| Gene umms.core.annotation.Gene.findLongestORF | ( |  | ) |  |

Here is the call graph for this function:

|  |  |  |  |  |  |
| --- | --- | --- | --- | --- | --- |
| static int [] umms.core.annotation.Gene.findLongestORF | ( | String | *sequence* | ) |  |

static

Here is the call graph for this function:

|  |  |  |  |  |  |
| --- | --- | --- | --- | --- | --- |
| boolean umms.core.annotation.Gene.geneSpanContains | ( | Gene | *gene* | ) |  |

Here is the call graph for this function:

Here is the caller graph for this function:

|  |  |  |  |
| --- | --- | --- | --- |
| int umms.core.annotation.Gene.genomicToGenomicPositionWithOffset | ( | int | *genomicPosition*, |
|  |  | int | *offset* |
|  | ) |  |  |

Get the genomic coordinate of the given position plus offset along the transcript

Parameters
:   |  |  |
    | --- | --- |
    | genomicPosition | Genomic position |
    | offset | Offset (positive gives result in 3' direction; negative gives result in 5' direction) |

Returns
:   The genomic position at the given transcript distance from the given position

Here is the call graph for this function:

|  |  |  |  |  |  |
| --- | --- | --- | --- | --- | --- |
| int umms.core.annotation.Gene.genomicToTranscriptPosition | ( | int | *genomicPosition* | ) |  |

Maps genomic coordinates to cDNA coordinates

Parameters
:   |  |  |
    | --- | --- |
    | genomicPosition | within transcript |

Returns
:   0-based position within oriented start of transcript

Here is the call graph for this function:

Here is the caller graph for this function:

|  |  |  |  |  |  |
| --- | --- | --- | --- | --- | --- |
| Annotation umms.core.annotation.Gene.get3PrimeExon | ( | int | *size* | ) |  |

Here is the call graph for this function:

|  |  |  |  |  |
| --- | --- | --- | --- | --- |
| Annotation umms.core.annotation.Gene.get3PrimeExon | ( |  | ) |  |

Here is the call graph for this function:

|  |  |  |  |  |  |
| --- | --- | --- | --- | --- | --- |
| int umms.core.annotation.Gene.get3PrimeGenomicDistance | ( | LightweightGenomicAnnotation | *annot* | ) |  |

Here is the call graph for this function:

|  |  |  |  |  |
| --- | --- | --- | --- | --- |
| Collection<? extends Annotation> umms.core.annotation.Gene.get3Utr | ( |  | ) |  |

Get the set of exons comprising the 3' UTR

Returns
:   the set of exons comprising the 3' UTR as a collection of Alignments objects

Here is the call graph for this function:

|  |  |  |  |  |
| --- | --- | --- | --- | --- |
| Annotation umms.core.annotation.Gene.get3UTR | ( |  | ) |  |

Here is the call graph for this function:

Here is the caller graph for this function:

|  |  |  |  |  |
| --- | --- | --- | --- | --- |
| Gene umms.core.annotation.Gene.get3UTRGene | ( |  | ) |  |

Here is the call graph for this function:

Here is the caller graph for this function:

|  |  |  |  |  |
| --- | --- | --- | --- | --- |
| Alignments umms.core.annotation.Gene.get3UtrIncludingIntrons | ( |  | ) |  |

Get the region of the gene span that lies 3' of the stop codon, including introns but not the stop codon itself

Returns
:   the region of the gene span that lies 3' of the stop codon as an Alignments object

Here is the call graph for this function:

Here is the caller graph for this function:

|  |  |  |  |  |
| --- | --- | --- | --- | --- |
| Annotation umms.core.annotation.Gene.get5PrimeExon | ( |  | ) |  |

Here is the call graph for this function:

|  |  |  |  |  |
| --- | --- | --- | --- | --- |
| Collection<? extends Annotation> umms.core.annotation.Gene.get5Utr | ( |  | ) |  |

Get the set of exons comprising the 5' UTR

Returns
:   the set of exons comprising the 5' UTR as a collection of Alignments objects

Here is the call graph for this function:

|  |  |  |  |  |
| --- | --- | --- | --- | --- |
| Annotation umms.core.annotation.Gene.get5UTR | ( |  | ) |  |

Here is the call graph for this function:

Here is the caller graph for this function:

|  |  |  |  |  |
| --- | --- | --- | --- | --- |
| Gene umms.core.annotation.Gene.get5UTRGene | ( |  | ) |  |

Here is the call graph for this function:

|  |  |  |  |  |
| --- | --- | --- | --- | --- |
| Annotation umms.core.annotation.Gene.get5UtrIncludingIntrons | ( |  | ) |  |

Get the region of the gene span that lies 5' of start codon, including introns and the start codon itself

Returns
:   the region of the gene span that lies 5' of start codon as an Alignments object

Here is the call graph for this function:

Here is the caller graph for this function:

|  |  |  |  |  |
| --- | --- | --- | --- | --- |
| Annotation umms.core.annotation.Gene.getAlignment | ( |  | ) |  |

Here is the call graph for this function:

Here is the caller graph for this function:

|  |  |  |  |  |
| --- | --- | --- | --- | --- |
| Iterator<Integer> umms.core.annotation.Gene.getAllPositions | ( |  | ) |  |

Get an iterator over all reference positions making up the gene

Returns
:   Iterator over all positions in the gene

Here is the call graph for this function:

|  |  |  |  |  |  |
| --- | --- | --- | --- | --- | --- |
| String umms.core.annotation.Gene.getAttribute | ( | String | *name* | ) |  |

Parameters
:   |  |  |
    | --- | --- |
    | name |  |

Returns
:   attribute value or 0 if the attribute is not defined.

|  |  |  |  |  |
| --- | --- | --- | --- | --- |
| Map umms.core.annotation.Gene.getAttributes | ( |  | ) |  |

Here is the caller graph for this function:

|  |  |  |  |  |
| --- | --- | --- | --- | --- |
| double umms.core.annotation.Gene.getBedScore | ( |  | ) |  |

Here is the caller graph for this function:

|  |  |  |  |  |
| --- | --- | --- | --- | --- |
| Gene umms.core.annotation.Gene.getCDS | ( |  | ) |  |

Here is the call graph for this function:

Here is the caller graph for this function:

|  |  |  |  |  |
| --- | --- | --- | --- | --- |
| int umms.core.annotation.Gene.getCDSEnd | ( |  | ) |  |

Here is the caller graph for this function:

|  |  |  |  |  |
| --- | --- | --- | --- | --- |
| Alignments umms.core.annotation.Gene.getCDSRegion | ( |  | ) |  |

Here is the call graph for this function:

Here is the caller graph for this function:

|  |  |  |  |  |
| --- | --- | --- | --- | --- |
| int umms.core.annotation.Gene.getCDSStart | ( |  | ) |  |

Here is the caller graph for this function:

|  |  |  |  |  |
| --- | --- | --- | --- | --- |
| String umms.core.annotation.Gene.getChrNum | ( |  | ) |  |

Return the chromosome number without the chr

Returns
:   the chromosome number without the chr

Here is the call graph for this function:

|  |  |  |  |  |
| --- | --- | --- | --- | --- |
| double umms.core.annotation.Gene.getCountScore | ( |  | ) |  |

|  |  |  |  |  |
| --- | --- | --- | --- | --- |
| Alignments [] umms.core.annotation.Gene.getExons | ( |  | ) |  |

Here is the call graph for this function:

|  |  |  |  |  |
| --- | --- | --- | --- | --- |
| Set<? extends Annotation> umms.core.annotation.Gene.getExonSet | ( |  | ) |  |

Here is the call graph for this function:

Here is the caller graph for this function:

|  |  |  |  |  |
| --- | --- | --- | --- | --- |
| double [] umms.core.annotation.Gene.getExonsScores | ( |  | ) |  |

|  |  |  |  |  |
| --- | --- | --- | --- | --- |
| IntervalTree<Annotation> umms.core.annotation.Gene.getExonTree | ( |  | ) |  |

Here is the call graph for this function:

|  |  |  |  |  |
| --- | --- | --- | --- | --- |
| String [] umms.core.annotation.Gene.getExtraFields | ( |  | ) |  |

Here is the caller graph for this function:

|  |  |  |  |  |  |
| --- | --- | --- | --- | --- | --- |
| String umms.core.annotation.Gene.getExtraFields | ( | int | *i* | ) |  |

|  |  |  |  |  |
| --- | --- | --- | --- | --- |
| Annotation umms.core.annotation.Gene.getFirstExon | ( |  | ) |  |

Returns
:   first exon in coordinate order

Here is the call graph for this function:

Here is the caller graph for this function:

|  |  |  |  |  |
| --- | --- | --- | --- | --- |
| Annotation umms.core.annotation.Gene.getFirstIntron | ( |  | ) |  |

Returns the first intron of this gene

Returns

Here is the call graph for this function:

|  |  |  |  |  |
| --- | --- | --- | --- | --- |
| int umms.core.annotation.Gene.getGappedSize | ( |  | ) |  |

|  |  |  |  |  |
| --- | --- | --- | --- | --- |
| int umms.core.annotation.Gene.getGenomicLength | ( |  | ) |  |

Here is the call graph for this function:

|  |  |  |  |  |
| --- | --- | --- | --- | --- |
| Gene umms.core.annotation.Gene.getIntrons | ( |  | ) |  |

Get introns

Returns
:   Gene object whose blocks are the introns, or null if no introns

Here is the call graph for this function:

Here is the caller graph for this function:

|  |  |  |  |  |
| --- | --- | --- | --- | --- |
| Alignments [] umms.core.annotation.Gene.getIntronsBlocks | ( |  | ) |  |

Here is the call graph for this function:

|  |  |  |  |  |
| --- | --- | --- | --- | --- |
| Collection<? extends Annotation> umms.core.annotation.Gene.getIntronSet | ( |  | ) |  |

Here is the call graph for this function:

Here is the caller graph for this function:

|  |  |  |  |  |
| --- | --- | --- | --- | --- |
| Gene umms.core.annotation.Gene.getIntronTranscript | ( |  | ) |  |

Here is the call graph for this function:

Here is the caller graph for this function:

|  |  |  |  |  |
| --- | --- | --- | --- | --- |
| Collection<Gene> umms.core.annotation.Gene.getIsoforms | ( |  | ) |  |

Here is the call graph for this function:

|  |  |  |  |  |
| --- | --- | --- | --- | --- |
| Annotation umms.core.annotation.Gene.getLastExon | ( |  | ) |  |

Returns
:   last exon in coordinate order

Here is the call graph for this function:

Here is the caller graph for this function:

|  |  |  |  |  |
| --- | --- | --- | --- | --- |
| Annotation umms.core.annotation.Gene.getLastIntron | ( |  | ) |  |

Here is the call graph for this function:

|  |  |  |  |  |
| --- | --- | --- | --- | --- |
| int umms.core.annotation.Gene.getMidpointGenomicCoords | ( |  | ) |  |

Here is the call graph for this function:

|  |  |  |  |  |
| --- | --- | --- | --- | --- |
| double umms.core.annotation.Gene.getNormalizedScore | ( |  | ) |  |

Here is the call graph for this function:

|  |  |  |  |  |  |
| --- | --- | --- | --- | --- | --- |
| double umms.core.annotation.Gene.getNumberPositions | ( | int | *rate* | ) |  |

Here is the call graph for this function:

|  |  |  |  |  |
| --- | --- | --- | --- | --- |
| int umms.core.annotation.Gene.getNumExons | ( |  | ) |  |

Here is the call graph for this function:

Here is the caller graph for this function:

|  |  |  |  |  |
| --- | --- | --- | --- | --- |
| Annotation umms.core.annotation.Gene.getOrientedLastExon | ( |  | ) |  |

Here is the call graph for this function:

|  |  |  |  |  |  |
| --- | --- | --- | --- | --- | --- |
| Gene umms.core.annotation.Gene.getOverlap | ( | Gene | *second* | ) |  |

Here is the call graph for this function:

Here is the caller graph for this function:

|  |  |  |  |  |  |
| --- | --- | --- | --- | --- | --- |
| Gene umms.core.annotation.Gene.getOverlap | ( | Collection< Gene > | *others* | ) |  |

Get all overlaps between this gene and any gene in the collection

Parameters
:   |  |  |
    | --- | --- |
    | others | The collection of genes to check for overlap |

Returns
:   A RefSeqGene object whose "exons" consist of overlapping intervals between this gene and the collection

Here is the call graph for this function:

|  |  |  |  |
| --- | --- | --- | --- |
| static Collection<Gene> umms.core.annotation.Gene.getOverlappers | ( | Collection< Gene > | *genes*, |
|  |  | String | *chr*, |
|  |  | int | *start*, |
|  |  | int | *end*, |
|  |  | Strand | *strand*, |
|  |  | boolean | *ignoreOrientation* |
|  | ) |  |  |

static

Get all genes overlapping a window

Parameters
:   |  |  |
    | --- | --- |
    | genes | The genes |
    | chr | Window chr |
    | start | Window start |
    | end | Window end |

Returns
:   All genes overlapping the window, not necessarily fully contained

Here is the call graph for this function:

|  |  |  |  |  |
| --- | --- | --- | --- | --- |
| double umms.core.annotation.Gene.getPercent3UTR | ( |  | ) |  |

Here is the call graph for this function:

|  |  |  |  |  |
| --- | --- | --- | --- | --- |
| double umms.core.annotation.Gene.getPercentCDS | ( |  | ) |  |

Here is the call graph for this function:

|  |  |  |  |  |  |
| --- | --- | --- | --- | --- | --- |
| Alignments umms.core.annotation.Gene.getPromoter | ( | int | *fudgeFactor* | ) |  |

Here is the call graph for this function:

Here is the caller graph for this function:

|  |  |  |  |
| --- | --- | --- | --- |
| Alignments umms.core.annotation.Gene.getPromoter | ( | int | *upstream*, |
|  |  | int | *downstream* |
|  | ) |  |  |

Here is the call graph for this function:

|  |  |  |  |  |
| --- | --- | --- | --- | --- |
| double umms.core.annotation.Gene.getPValue | ( |  | ) |  |

|  |  |  |  |  |
| --- | --- | --- | --- | --- |
| double umms.core.annotation.Gene.getRPKM | ( |  | ) |  |

|  |  |  |  |  |
| --- | --- | --- | --- | --- |
| String umms.core.annotation.Gene.getSAMString | ( |  | ) |  |

|  |  |  |  |  |
| --- | --- | --- | --- | --- |
| String umms.core.annotation.Gene.getSequence | ( |  | ) |  |

|  |  |  |  |  |  |
| --- | --- | --- | --- | --- | --- |
| Sequence umms.core.annotation.Gene.getSequence | ( | Sequence | *chrSequence* | ) |  |

Here is the call graph for this function:

|  |  |  |  |
| --- | --- | --- | --- |
| String umms.core.annotation.Gene.getSequence | ( | Sequence | *chr2*, |
|  |  | boolean | *repeatMask*, |
|  |  | boolean | *stranded* |
|  | ) |  | throws Exception |

|  |  |  |  |  |
| --- | --- | --- | --- | --- |
| Sequence umms.core.annotation.Gene.getSequenceObject | ( |  | ) |  |

Here is the call graph for this function:

|  |  |  |  |  |
| --- | --- | --- | --- | --- |
| int umms.core.annotation.Gene.getSize | ( |  | ) |  |

Returns
:   same as size();

Implements umms.core.annotation.Annotation.

Here is the call graph for this function:

Here is the caller graph for this function:

|  |  |  |  |  |
| --- | --- | --- | --- | --- |
| Collection<? extends Annotation> umms.core.annotation.Gene.getSortedAndUniqueExons | ( |  | ) |  |

Here is the call graph for this function:

Here is the caller graph for this function:

|  |  |  |  |  |
| --- | --- | --- | --- | --- |
| ArrayList<Integer> umms.core.annotation.Gene.getSpliceJunctionCoordinates | ( |  | ) | throws Exception |

Here is the call graph for this function:

Here is the caller graph for this function:

|  |  |  |  |  |
| --- | --- | --- | --- | --- |
| IntervalTree<Integer> umms.core.annotation.Gene.getSpliceJunctionCoordinatesTree | ( |  | ) | throws Exception |

Here is the call graph for this function:

|  |  |  |  |  |
| --- | --- | --- | --- | --- |
| Gene umms.core.annotation.Gene.getStartCodon | ( |  | ) |  |

Here is the call graph for this function:

|  |  |  |  |  |
| --- | --- | --- | --- | --- |
| String umms.core.annotation.Gene.getString | ( |  | ) |  |

Here is the call graph for this function:

|  |  |  |  |  |
| --- | --- | --- | --- | --- |
| int umms.core.annotation.Gene.getTranscriptLength | ( |  | ) |  |

Here is the call graph for this function:

|  |  |  |  |  |  |
| --- | --- | --- | --- | --- | --- |
| Collection<GeneWindow> umms.core.annotation.Gene.getWindows | ( | int | *windowSize* | ) |  |

Here is the caller graph for this function:

|  |  |  |  |
| --- | --- | --- | --- |
| Collection<GeneWindow> umms.core.annotation.Gene.getWindows | ( | int | *windowSize*, |
|  |  | int | *stepSize*, |
|  |  | int | *start* |
|  | ) |  |  |

Here is the call graph for this function:

|  |  |  |  |  |
| --- | --- | --- | --- | --- |
| boolean umms.core.annotation.Gene.hasCDS | ( |  | ) |  |

Here is the caller graph for this function:

|  |  |  |  |  |
| --- | --- | --- | --- | --- |
| int umms.core.annotation.Gene.hashCode | ( |  | ) |  |

Here is the call graph for this function:

|  |  |  |  |  |  |
| --- | --- | --- | --- | --- | --- |
| void umms.core.annotation.Gene.initFromGene | ( | Gene | *gene* | ) |  |

protected

Here is the call graph for this function:

Here is the caller graph for this function:

|  |  |  |  |  |
| --- | --- | --- | --- | --- |
| boolean umms.core.annotation.Gene.isCodingGene | ( |  | ) |  |

Whether the gene is coding or noncoding

Returns
:   True if the cds exists and has size at least 3

Here is the call graph for this function:

|  |  |  |  |  |  |
| --- | --- | --- | --- | --- | --- |
| boolean umms.core.annotation.Gene.isFullyCompatible | ( | Gene | *iso* | ) |  |

Here is the call graph for this function:

|  |  |  |  |  |  |
| --- | --- | --- | --- | --- | --- |
| boolean umms.core.annotation.Gene.isInExon | ( | int | *snp* | ) |  |

|  |  |  |  |  |
| --- | --- | --- | --- | --- |
| boolean umms.core.annotation.Gene.isNegativeStrand | ( |  | ) |  |

Implements umms.core.annotation.Annotation.

Here is the call graph for this function:

Here is the caller graph for this function:

|  |  |  |  |  |  |
| --- | --- | --- | --- | --- | --- |
| static boolean umms.core.annotation.Gene.isStopCodon | ( | String | *currentCodon* | ) |  |

static

Here is the caller graph for this function:

|  |  |  |  |  |  |
| --- | --- | --- | --- | --- | --- |
| int umms.core.annotation.Gene.numOfCompatibleIntrons | ( | Gene | *g* | ) |  |

Here is the call graph for this function:

Here is the caller graph for this function:

|  |  |  |  |  |  |
| --- | --- | --- | --- | --- | --- |
| int umms.core.annotation.Gene.numOverlappingExons | ( | Gene | *iso* | ) |  |

Here is the call graph for this function:

|  |  |  |  |  |  |
| --- | --- | --- | --- | --- | --- |
| boolean umms.core.annotation.Gene.overlaps | ( | Gene | *other* | ) |  |

Whether an exon of this gene overlaps an exon of other gene and the two genes have the same orientation

Parameters
:   |  |  |
    | --- | --- |
    | other | Other gene |

Returns
:   Whether genes have same orientation and at least one overlapping exon

Here is the caller graph for this function:

|  |  |  |  |
| --- | --- | --- | --- |
| boolean umms.core.annotation.Gene.overlaps | ( | Gene | *other*, |
|  |  | boolean | *ignoreOrientation* |
|  | ) |  |  |

Whether an exon of this gene overlaps an exon of other gene

Parameters
:   |  |  |
    | --- | --- |
    | other | Other gene |
    | ignoreOrientation | Ignore orientation. If set to false, orientations must be equal or at least one orientation must be unknown. |

Returns
:   Whether genes overlap at the exon level

Here is the call graph for this function:

|  |  |  |  |
| --- | --- | --- | --- |
| boolean umms.core.annotation.Gene.overlaps | ( | Gene | *other*, |
|  |  | double | *minPctOverlap* |
|  | ) |  |  |

Returns true if this and the other gene overlap but at least minPctOverlap

Parameters
:   |  |  |
    | --- | --- |
    | other | The other gene with which overlap is checked |
    | minPctOverlap | minimum percent of overlap |

Returns

Here is the call graph for this function:

|  |  |  |  |  |  |
| --- | --- | --- | --- | --- | --- |
| boolean umms.core.annotation.Gene.overlaps2 | ( | LightweightGenomicAnnotation | *next* | ) |  |

Here is the call graph for this function:

|  |  |  |  |  |  |
| --- | --- | --- | --- | --- | --- |
| boolean umms.core.annotation.Gene.overlapsExon | ( | Annotation | *gen* | ) |  |

Here is the call graph for this function:

|  |  |  |  |  |  |
| --- | --- | --- | --- | --- | --- |
| boolean umms.core.annotation.Gene.overlapsExon | ( | LightweightGenomicAnnotation | *exon* | ) |  |

|  |  |  |  |  |  |
| --- | --- | --- | --- | --- | --- |
| boolean umms.core.annotation.Gene.overlapsGene | ( | Collection< Gene > | *others* | ) |  |

Whether this gene overlaps any gene in the collection at the exon level

Parameters
:   |  |  |
    | --- | --- |
    | others | The genes to check for overlap |

Returns
:   True iff this gene overlaps at least one gene in the collection at the exon level

Here is the call graph for this function:

|  |  |  |  |  |  |
| --- | --- | --- | --- | --- | --- |
| boolean umms.core.annotation.Gene.overlapsGene | ( | Gene | *gen* | ) |  |

Here is the call graph for this function:

|  |  |  |  |  |  |
| --- | --- | --- | --- | --- | --- |
| boolean umms.core.annotation.Gene.overlapsGeneInAnyOrientation | ( | Gene | *otherGene* | ) |  |

Here is the call graph for this function:

|  |  |  |  |
| --- | --- | --- | --- |
| boolean umms.core.annotation.Gene.overlapsStranded | ( | Gene | *other*, |
|  |  | double | *minPctOverlap* |
|  | ) |  |  |

Returns true if this and the other gene overlap but at least minPctOverlap

Parameters
:   |  |  |
    | --- | --- |
    | other | The other gene with which overlap is checked |
    | minPctOverlap | minimum percent of overlap |

Returns

Here is the call graph for this function:

|  |  |  |  |  |  |
| --- | --- | --- | --- | --- | --- |
| Double umms.core.annotation.Gene.percentGenomeOverlapping | ( | Gene | *gene2* | ) |  |

Here is the call graph for this function:

|  |  |  |  |  |  |
| --- | --- | --- | --- | --- | --- |
| double umms.core.annotation.Gene.percentOverlapping | ( | Gene | *gene2* | ) |  |

Here is the call graph for this function:

Here is the caller graph for this function:

|  |  |  |  |  |  |
| --- | --- | --- | --- | --- | --- |
| void umms.core.annotation.Gene.setBedScore | ( | double | *d* | ) |  |

|  |  |  |  |  |  |
| --- | --- | --- | --- | --- | --- |
| void umms.core.annotation.Gene.setCDS | ( | Annotation | *cdsRegion* | ) |  |

|  |  |  |  |  |  |
| --- | --- | --- | --- | --- | --- |
| void umms.core.annotation.Gene.setCDSEnd | ( | int | *end* | ) |  |

|  |  |  |  |
| --- | --- | --- | --- |
| void umms.core.annotation.Gene.setCDSRegion | ( | int | *start*, |
|  |  | int | *end* |
|  | ) |  |  |

Set coding region coordinates (includes start codon but not stop codon)

Parameters
:   |  |  |
    | --- | --- |
    | start | start position |
    | end | end position |

Here is the call graph for this function:

|  |  |  |  |  |  |
| --- | --- | --- | --- | --- | --- |
| void umms.core.annotation.Gene.setCDSStart | ( | int | *start* | ) |  |

|  |  |  |  |  |  |
| --- | --- | --- | --- | --- | --- |
| void umms.core.annotation.Gene.setCountScore | ( | double | *score* | ) |  |

|  |  |  |  |  |  |
| --- | --- | --- | --- | --- | --- |
| void umms.core.annotation.Gene.setExtraFields | ( | double[] | *d* | ) |  |

Here is the call graph for this function:

Here is the caller graph for this function:

|  |  |  |  |
| --- | --- | --- | --- |
| void umms.core.annotation.Gene.setExtraFields | ( | double | *val*, |
|  |  | int | *i* |
|  | ) |  |  |

|  |  |  |  |  |  |
| --- | --- | --- | --- | --- | --- |
| void umms.core.annotation.Gene.setExtraFields | ( | String[] | *extraData* | ) |  |

|  |  |  |  |  |  |
| --- | --- | --- | --- | --- | --- |
| void umms.core.annotation.Gene.setOrientation | ( | String | *orientation* | ) |  |

Here is the call graph for this function:

|  |  |  |  |  |  |
| --- | --- | --- | --- | --- | --- |
| void umms.core.annotation.Gene.setSAMString | ( | String | *sam* | ) |  |

|  |  |  |  |  |  |
| --- | --- | --- | --- | --- | --- |
| void umms.core.annotation.Gene.setSequence | ( | String | *seq* | ) |  |

Here is the caller graph for this function:

|  |  |  |  |  |  |
| --- | --- | --- | --- | --- | --- |
| void umms.core.annotation.Gene.setSequenceFromChromosome | ( | Sequence | *chrSequence* | ) |  |

Here is the call graph for this function:

|  |  |  |  |  |  |
| --- | --- | --- | --- | --- | --- |
| Gene umms.core.annotation.Gene.takeUnion | ( | Gene | *other* | ) |  |

Here is the call graph for this function:

|  |  |  |  |  |
| --- | --- | --- | --- | --- |
| String umms.core.annotation.Gene.toBED | ( |  | ) |  |

Implements umms.core.annotation.Annotation.

Here is the caller graph for this function:

|  |  |  |  |
| --- | --- | --- | --- |
| String umms.core.annotation.Gene.toBED | ( | int | *r*, |
|  |  | int | *g*, |
|  |  | int | *b* |
|  | ) |  |  |

Implements umms.core.annotation.Annotation.

Here is the call graph for this function:

|  |  |  |  |  |  |
| --- | --- | --- | --- | --- | --- |
| String umms.core.annotation.Gene.toBED | ( | boolean | *useExtraFields* | ) |  |

Here is the call graph for this function:

|  |  |  |  |
| --- | --- | --- | --- |
| String umms.core.annotation.Gene.toBED | ( | boolean | *useExtraFields*, |
|  |  | int | *r*, |
|  |  | int | *g*, |
|  |  | int | *b* |
|  | ) |  |  |

Here is the call graph for this function:

|  |  |  |  |  |
| --- | --- | --- | --- | --- |
| String umms.core.annotation.Gene.toBEDwithBedScore | ( |  | ) |  |

Here is the call graph for this function:

|  |  |  |  |
| --- | --- | --- | --- |
| String umms.core.annotation.Gene.toCufflinksGTF | ( | String | *source*, |
|  |  | String | *geneName*, |
|  |  | String | *transcriptName*, |
|  |  | String | *nonParsedAttrs* |
|  | ) |  |  |

Here is the call graph for this function:

|  |  |  |  |  |  |
| --- | --- | --- | --- | --- | --- |
| String umms.core.annotation.Gene.toGTF | ( | String | *source* | ) |  |

Here is the call graph for this function:

|  |  |  |  |
| --- | --- | --- | --- |
| String umms.core.annotation.Gene.toGTF | ( | String | *source*, |
|  |  | String | *geneName*, |
|  |  | String | *transcriptName* |
|  | ) |  |  |

Here is the call graph for this function:

|  |  |  |  |  |
| --- | --- | --- | --- | --- |
| String umms.core.annotation.Gene.toRefSeq | ( |  | ) |  |

Here is the call graph for this function:

|  |  |  |  |  |
| --- | --- | --- | --- | --- |
| String umms.core.annotation.Gene.toSAM | ( |  | ) |  |

Here is the call graph for this function:

Here is the caller graph for this function:

|  |  |  |  |  |  |
| --- | --- | --- | --- | --- | --- |
| String umms.core.annotation.Gene.toSAM | ( | String | *originalSAMLine* | ) |  |

Here is the call graph for this function:

|  |  |  |  |
| --- | --- | --- | --- |
| String umms.core.annotation.Gene.toSAM | ( | String | *originalSAMLine*, |
|  |  | boolean | *flip* |
|  | ) |  |  |

Here is the call graph for this function:

|  |  |  |  |  |
| --- | --- | --- | --- | --- |
| String umms.core.annotation.Gene.toString | ( |  | ) |  |

Here is the caller graph for this function:

|  |  |  |  |  |
| --- | --- | --- | --- | --- |
| String umms.core.annotation.Gene.toUCSC | ( |  | ) |  |

Returns
:   chrX:start-end

Implements umms.core.annotation.Annotation.

Here is the call graph for this function:

Here is the caller graph for this function:

|  |  |  |  |  |  |
| --- | --- | --- | --- | --- | --- |
| int umms.core.annotation.Gene.transcriptToGenomicPosition | ( | int | *transcriptPosition* | ) |  |

Here is the call graph for this function:

Here is the caller graph for this function:

|  |  |  |  |
| --- | --- | --- | --- |
| Gene umms.core.annotation.Gene.transcriptToGenomicPosition | ( | int | *startPosOnTranscript*, |
|  |  | int | *endPosOnTranscript* |
|  | ) |  |  |

Convert an interval in transcriptome space to genome space

Parameters
:   |  |  |
    | --- | --- |
    | startPosOnTranscript | Start position in transcriptome space |
    | endPosOnTranscript | End position in transcriptome space |

Returns
:   The interval (possibly spliced) in genome space

Here is the call graph for this function:

|  |  |  |  |
| --- | --- | --- | --- |
| GeneWindow umms.core.annotation.Gene.trimAbsolute | ( | int | *alignmentStart*, |
|  |  | int | *alignmentEnd* |
|  | ) |  |  |

Here is the call graph for this function:

Here is the caller graph for this function:

|  |  |  |  |
| --- | --- | --- | --- |
| GeneWindow umms.core.annotation.Gene.trimGene | ( | int | *relativeStart*, |
|  |  | int | *relativeEnd* |
|  | ) |  |  |

Trims the gene in relative space Returns a GeneWindow with pointer to the original

Here is the call graph for this function:

Here is the caller graph for this function:

## Member Data Documentation

final Pattern umms.core.annotation.Gene.START\_CODON\_PATRN = Pattern.compile("ATG", Pattern.CASE\_INSENSITIVE)

static

final String [] umms.core.annotation.Gene.STOP\_CODONS = {"TAG", "TAA", "TGA"}

static

---

The documentation for this class was generated from the following file:

- java/umms/core/annotation/Gene.java


---

Generated on Fri Aug 15 2014 15:11:09 for ESAT by  

 1.8.7
